# Divergent Effectiveness of Multispecies Probiotic Preparations on Intestinal Microbiota Structure Depends on Metabolic Properties

**DOI:** 10.3390/nu11020325

**Published:** 2019-02-02

**Authors:** Michele Biagioli, Daniela Capobianco, Adriana Carino, Silvia Marchianò, Chiara Fiorucci, Patrizia Ricci, Eleonora Distrutti, Stefano Fiorucci

**Affiliations:** 1 Dipartimento di Scienze Biomediche e Chirurgiche, Università di Perugia, 06123 Perugia, Italy; michele.biagioli@unipg.it (M.B.); adriana.carino@unipg.it (A.C.); silvia4as@hotmail.it (S.M.); chiara.fiorucci125@gmail.com (C.F.); patrizia.ricci@unipg.it (P.R.); 2 Department of Public Health and Infectious Diseases, University of Rome La Sapienza, 00185 Rome, Italy; daniela.capobianco@uniroma1.it; 3 SC di Gastroenterologia ed Epatologia, Azienda Ospedaliera di Perugia, 06100 Perugia, Italy; eleonora.diastrutti@katamail.com

**Keywords:** probiotics, inflammatory bowel disease, animal studies, macrophage, regulatory T (Treg) cells, lactic acid, short-chain fatty acids (SCFA), intestinal microbiota

## Abstract

A growing body of evidence suggests that probiotic functionality is not accurately predicted by their taxonomy. Here, we have set up a study to investigate the effectiveness of two probiotic formulations containing a blend of seven bacterial species in modulating intestinal inflammation in two rodent models of colitis, induced by treating mice with 2,4,6-Trinitrobenzenesulfonic acid (TNBS) or dextran sodium sulfate (DSS). Despite the taxonomy of the bacterial species in the two probiotic formulations being similar, only one preparation (Blend 2-Vivomixx) effectively attenuated the development of colitis in both models. In the TNBS model of colitis, Blend 2 reduced the expression of pro-inflammatory genes while increasing the production of anti-inflammatory cytokines, promoting the expansion M2 macrophages and the formation of IL-10-producing Treg cells in the colon’s *lamina propria*. In the DSS model of colitis, disease attenuation and Treg formation was observed only in mice administered with Blend 2, and this effect was associated with intestinal microbiota remodeling and increased formation of lactate, butyrate, and propionate. None of these effects were observed in mice administered with Blend 1 (VSL#3). In summary, we have shown that two probiotic mixtures obtained by combining taxonomically similar species produced with different manufacturing methods exert divergent effects in mouse models of colitis.

## 1. Introduction

Probiotics are defined as: “live microorganisms which when administered in adequate amounts confer a health benefit on the host” [1,2]. While they have a number of beneficial health effects in common, it is acknowledged that some functional activities are strain-specific. Consequently, there is a need for rigorous strain-to-strain comparison of the same species in regard to therapeutic activity and efficacy [1,2]. Since the functional activity of taxonomically similar or even identical strains manufactured by different producers varies, the health-promoting properties of a specific formulation cannot be extrapolated from one formulation to another without in vitro and in vivo comparative testing [3,4]. Fittingly, it is well established that the immune-modulatory effects of two widely investigated Lactobacilli, *L. plantarum* and *L. gasseri*, i.e., their ability to reciprocally modulate IL-10/IL-12 production in cell cultures, changes widely according to culture conditions and media composition [5,6]. 

Recently, a cluster of studies have reported that different lots of a commercially available multistrain probiotic preparation, available in Europe and North America under the brand name of VSL#3^®^, show different biochemical and immunological properties in vitro and exert variable activity in rodent models of intestinal inflammation, depending on the manufacturing site [7,8,9,10], which suggests that the health-promoting properties of these preparations are affected by the manufacturing processes [8,11,12]. 

Taken together, these data support the notion that culture conditions, in addition to strain taxonomy, impact on the probiotic properties of genetically similar bacterial strains and that different manufacturing sources might result in probiotic preparations with profound functional variability despite identical taxonomy [7,10]. Together, these studies raise the need for a more appropriate definition of the functional properties of the bacterial strains commonly commercialized as probiotics either alone or blended.

In recent years, two different probiotic blends that contain the same mixture of seven taxonomically identical bacterial species have been made commercially available (i.e., VSL#3 and Vivomixx). According to the box labeling, the two blends are mixture of four strains of *Lactobacilli*, three strains of *Bifidobacteria*, and one strain of *Streptococcus thermophilus*. However, the relative proportion of each component in the final mixture and manufacturing conditions are not reported. 

Because both VSL#3 and Vivomixx are recommended for use at the same daily dose, and both claim similar health promoting activities, we have set up a study to investigate the effectiveness of the two formulations in modulating the structure of the intestinal microbiota and markers of inflammation in two rodent models of colitis. Additionally, since some of the beneficial effects exerted by probiotics are mediated by their metabolites, including lactate and short-chain fatty acids (SCFA) [13], we have performed an in vitro/in vivo study to test whether the two mixtures generate similar amounts of lactate and SCFAs.

## 2. Materials and Methods 

### 2.1. Probiotics

Two different formulations of probiotics were used in this study: VSL#3^®^, indicated as Blend 1, and Vivomixx^®^) indicated as Blend 2. Both lots were commercially available in pharmacies in Italy. The lot numbers and expiration dates were the following: VSL#3^®^ lot 705006, expiration 05/2019, and Vivomixx^®^ lot 1719104, expiration 31/07/2019. The list of strains as they appear on their respective commercial packaging is as follow: *Streptococcus thermophilus* BT01, *Lactobacillus plantarum* BP06, *Bifidobacterium breve* BB02, *Bifidobacterium longum* BL03, *Bifidobacterioum infantis* BI04, *Lactobacillus acidophilus* BA05, *Lactobacillus paracasei* BP07, *Lactobacillus debrueckii subsp. bulgaricus* BD08, for VSL#3^®^ and *Streptococcus thermophilus* DSM24731^®^, *Lactobacillus plantarum* DSM24730^®^, *Bifidobacterium breve* DSM24732^®^, *Lactobacillus paracasei* DSM24733^®^, *Lactobacillus delbrueckii subsp. bulgaricus* DSM24734^®^, *Lactobacillus acidophilus* DSM 24735^®^, *Bifidobacterium longum* DSM24736^®^, *Bifidobacterium infantis* DSM24737^®^, for Vivomixx^®^. The different code names indicate that the bacteria have different sources and are registered in different cell repositories. The two batches were maintained according to the manufacturer instructions until used. 

### 2.2. Bacterial Growth Conditions and Viability Test

Microorganisms from lyophilized probiotic products stored at 4 °C were inoculated onto de Man-Rogosa-Sharpe (MRS) broth (Thermo Scientific™, Waltham, MA, USA) containing 0.05% l-cysteine-HCl (Sigma-Aldrich, St. Louis, MO, USA). Viable microorganisms were determined by plating serial 10-fold dilutions onto MRS-agar (Thermo Scientific™) containing 0.05% l-cysteine-HCl. Tests were performed in duplicate. Colonies were enumerated after incubation of plates at 37 °C for 48 h under anaerobic conditions in Anaerogen system (Thermo Scientific™). Individual strains from Blend 2 were provided by Mendes S.A.-Via Giacometti, 1, 6900 Lugano, Switzerland).

### 2.3. Residual Carbohydrate Determination and Lactic Acid Production

Ability of lactic acid bacteria to ferment carbohydrates was determined using MRS broth (basal MRS) or MRS supplemented with filter-sterilized fructose. Microorganisms from lyophilized probiotic products were suspended in basal MRS at a concentration of 10^10^ viable bacteria/ml. The stock suspension was then diluted to 10^8^ viable bacteria/ml in basal MRS broth or MRS supplemented with 0.5 and 2% fructose and cultured at 37 °C for 21 h under anaerobic conditions. Cells were removed by centrifugation (7600× *g*, 15 min) and supernatants stored at −20 °C for subsequent analyses. Residual glucose and fructose from cell-free culture samples were determined using Enzytec™ Liquid d-Glucose/d-Fructose (R-Biopharm AG, Darmstadt, Germany). Lactic acid production was evaluated with a commercial kit for the determination of D- and L-lactic acid (Test-Combination, UV-method; Boehringer Mannheim/R-Biopharm, Darmstadt, Germany). The NADH increase, stoichiometric to the amount of d- and l-lactic acid, was determined by absorbance at 340 nm, according to manufacturer instruction. Fermentations were carried out in triplicate as independent experiments. The reported data correspond to the median of at least three measurements from three independent experiments. Viable counts at the beginning and the end of each experiment were determined as CFU/mL.

### 2.4. Animals and Colitis Protocols

Balb/c mice were obtained from Charles River (Lecco, Italy). The colonies were maintained in the animal facility of the University of Perugia. The mice were housed under controlled temperatures (22 °C) and photoperiods (12:12-hour light/dark cycle), allowed unrestricted access to standard mouse chow and tap water, and allowed to acclimate to these conditions for at least 5 days before inclusion in an experiment. The study was conducted in agreement with the Italian law and the protocol was approved by an ethical committee of the University of Perugia and by a National Committee of Italian Ministry of Health permit n° 1126/2016-PR. The health and body conditions of the animals were monitored daily by the veterinarian in the animal facility. Only male mice were used in each experiment. The study protocol caused minor suffering; however, animals losing more than 25% of their initial body weight were euthanized. In the first model, colitis was induced in Balb/c mice using 5% DSS (DSS: Dextran Sulfate, Sodium Salt of Affymetrix USA, molecular mass 40–50 kDa) in drinking water for 8 consecutive days. We used 8–12 animals for each experimental group. Animals were monitored daily. In the second model, the model of colitis induced by 2,4,6-trinitrobenzenesulfonic acid (TNBS), Balb/c mice were fasted for 12 h (day^−1^). The day after (day 0), mice were anesthetized, and a 3.5 F catheter inserted into the colon such that the tip was 4 cm proximal to the anus. To induce colitis, 1 mg of TNBS (Sigma Chemical Co., St Louis, MO, USA) in 50% ethanol was administered via a catheter into the lumen using a 1 mL syringe (injection volume of 100 μL); control mice received 50% ethanol alone. We used 5–7 animals for each experimental group. Animals were monitored daily for 4 days. At the end of the experiments, the surviving mice were sacrificed, blood samples collected by cardiac puncture, and the colon was excised, weighed, and evaluated for macroscopic damage. In some groups of mice, we also administered Blend 1 or Blend 2 by oral gavage at the concentration of 50 × 10^9^ probiotic cfu/kg of body weight dissolved in saline solution [7,14,15]. Both treatments were administered daily from day 0 to the day of sacrifice. In both animal models, the severity of colitis was measured each day for each mouse by assessing body weight, fecal blood, and stool consistency. Each parameter was scored from 0 to 4 as described previously [7]. At the end of the study, surviving animals were used for different biochemical and functional analyses as detailed in each Figure, assuring that at least 4 animals for each experimental group were available for each determination.

### 2.5. Histology

Samples of distal colon (2–3 cm from the anus) were fixed in buffered formalin, cut into 5-µm-thick sections (150 µm between each section, four to eight per fragment per colon), and stained with Hematoxylin and eosin (H&E).

### 2.6. Isolation of Lamina Propria Cells

The cells were isolated from the colon *lamina propria* using the Lamina Propria Dissociation Kit (Miltenyi Biotec, Bergisch Gladbach, Germania; 130-097-401), according to the instructions.

### 2.7. Flow Cytometry

Flow cytometry analyses were carried out using a two-laser standard configuration BD FACSVia™ flow cytometry system. We analyzed 6–10 mice for each experimental group. Data were analyzed using FlowJo software (TreeStar, Ashland, OR, USA). The gates were set using fluorescence minus one (FMO,) control strategy. FMO controls are samples that include all conjugated Abs present in the test samples except one. The channel in which the conjugated Ab is missing is the one for which the FMO provides a gating control. The following mAbs were used: CD4 PerCp-Cy5.5 (RM4-5, eBioscience, San Diego, CA, USA); CD11b Pe-Cy7 (M1/70, eBioscience); Gr1 PE (RB6-8C5, BioLegend); IL-10 FITC (JES5-16E3, eBioscience) and FoxP3 APC (FJK-16s, eBioscience).

### 2.8. Reverse Transcription of mRNA and Real-Time PCR

Colon samples were immediately frozen in liquid nitrogen and stored at −80 °C until used, mechanically homogenated with the aid of a pestle, and the obtained materials re-suspended in 1 mL of Trizol (Thermo Scientific™). The RNA was extracted according to the manufacturer’s protocol. After purification from genomic DNA using DNase-I treatment (Thermo Scientific™), 1 µg of RNA from each sample was reverse-transcribed using random hexamer primers with Superscript-II (Thermo Scientific™) in a 20 μL reaction volume; 10 ng cDNA were amplified in a 20 μL solution containing 200 nM of each primer and 10 μL of SYBR Select Master Mix (Thermo Scientific™). All reactions were performed in triplicate, and the thermal cycling conditions were as follows: 3 min at 95 °C, followed by 40 cycles of 95 °C for 15 s, 56 °C for 20 s, and 72 °C for 30 s, using a Step One Plus machine (Applied Biosystem). The relative mRNA expression was calculated accordingly to the 2^(−ΔCt)^ method comparing the expression of different genes to that of GAPDH housekeeping. Primers were designed using the software PRIMER3 (http://frodo.wi.mit.edu/primer3/) using published data obtained from the NCBI database. For each drawn primer we verified the correct alignment in the target gene using UCSC genome browser (https://genome.ucsc.edu/cgi-bin/hgBlat) and we verified the efficiency in the laboratory using cDNA at known concentration in scalar dilutions. Alternatively, for some genes the TaqMan probes (Thermo Scientific™) were used, with TaqMan GEX Master Mix (Thermo Scientific™). The primer used was conducted as follows (forward and reverse): Gapdh (for CTGAGTATGTCGTGGAGTCTAC; rev GTTGGTGGTGCAGGATGCATTG; Ifn-γ (for GCTTTGCAGCTCTTCCTCAT; rev ATCCTTTTGCCAGT), Tnf-α (for CCACCACGCTCTTCTGTCTA; rev AGGGTCTGGGCCATAGAACT), Il-6 (for CTTCACAAGTCGGAGGCTTA; rev TTCTGCAAGTGCATCATCGT), Il-1β (for GCTGAAAGCTCTCCACCTCA; rev AGGCCACAGGTATTTTGTCG), Tgf-β (for TTGCTTCAGCTCCACAGAGA; rev TGGTTGTAGAGGGCAAGGAC), Il-10 (for CCCAGAAATCAAGGAGCATT; rev CTCTTCACCTGCTCCACTGC), FoxP3 (for TCTTCGAGGAGCCAGAAGAG; rev AGCTCCCAGCTTCTCCTTTT). We also used the following TaqMan probes: Cd38 (Mm01220906_m1 Thermo Scientific™) and c-myc (Mm00487804_m1 Thermo Scientific™).

### 2.9. LC/ESI/MS Analysis of SCFAs in Feces

SCFAs in fecal samples of mice were measured using liquid chromatography-mass spectrometry as described by [16] using a LTQ-FT system (Thermo Scientific™) equipped with a Phenomenex Synergi Polar-RP column (Phenomenex, Torrance, CA, USA). The results are calculated in nmol/mg of fecal sample.

### 2.10. Metagenomics

*DNA extraction.* The microbial DNA was purified from mouse stool samples (4 mice for each experimental group), using the PureLink Microbiome DNA Purification Kit (Thermo Scientific™), according the manufacturer’s instructions. Briefly, approximately 100 mg of mouse stool were weighed and transferred to the bead tube and mixed thoroughly with 700 µL of S1-lysis buffer and 100 µL of S2-lysis enhancer to create a homogeneous sample and incubated at 65 °C for 10 min. The bead tubes were homogenized for 10 minutes at maximum speed on the horizontal vortex mixer, then centrifuged at 14,000× *g* for 5 min, and 400 µL of supernatant was transferred to a clean micro-centrifuge tube and vortexed immediately with 250 µL of S3-cleanup buffer. After 2 min of centrifugation, 500 µL of supernatant was transferred in a new Eppendorf and mixed with 900 µL of S4-binding buffer. Then 700 µL of sample mixture were loaded onto a spin column tube and centrifuged at 14,000× *g* for 1 min (2×). The spin column was then washed with 500 µL of S5-wash buffer and the flow-through was discarded. Finally, the spin column was placed in a clean tube, and the purified DNA was eluted with 100 µL of S6-elution buffer. The isolated DNA was quantified with a Qubit dsDNA HS Assay Kit on Qubit 3.0 fluorometer (Thermo Scientific™), according the manufacturer’s instructions, and then stored at −20 °C. 

*16S rRNA sequencing*. Sequencing was performed using an Ion 16S Metagenomics Kit (Thermo Scientific™) on the Ion Torrent S5 platform (Thermo Scientific™). Briefly, 3 ng of DNA was subjected to amplification of 16S rRNA libraries using two primer pools to amplify seven hypervariable regions of bacterial 16S rRNA. Primers were partially digested and barcoded adapters (Ion Xpress Barcode Adapters 1-16 Kit) ligated to the amplicons, using the Ion Plus Fragment Library Kit (Thermo Scientific™), purified using Agencourt AMPure XP beads (Beckman Coulter, Brea, CA, USA) according to the manufacturer’s protocol, and stored at −20 °C until further processing. The concentration of each 16S library was determined by qPCR using the Ion Library Quantitation Kit and a Qubit 3.0 fluorometer (Thermo Scientific™). The library was diluted to ~100 pM prior to template preparation. Template preparation of the barcoded libraries was performed using the Ion Chef and the Ion S5 System (Thermo Scientific™). A maximum of 16 barcoded 16S samples were sequenced on a Ion 520 chip (Thermo Scientific™) using the Ion 510 & Ion 520 & Ion 530 Kit-Chef (Thermo Scientific™) according to the manufacturer’s instructions. 

*Metagenomics analysis*. Automated analysis, annotation, and taxonomical assignment were generated using Ion Reporter Software—Metagenomics Workflow (Ion Reporter 5.10.2.0, Thermo Scientific™). The Ion Reporter Software enables the rapid identification (at genus or species level) of microbes present in each sample, using both curated Greengenes and premium curated MicroSEQ ID 16S rRNA reference databases. The Ion Reporter metagenomics workflow also provides primer information, classification information, percent ID, and mapping information. 

*Data visualization and statistical analyses of taxonomy.* Data visualization and statistical analyses were performed using Krona and QIIME™ analysis software, and related packages were used for diversity and correlation analyses. Principal coordinates analysis (PCoA) was conducted with identified reads/OTUs using classical multidimensional scaling (Bray-Curtis) to analyze distribution of dissimilarities and analysis of variance using abundance data. 

### 2.11. Statistical analysis

The ANOVA followed by a non-parametric Mann-Whitney U test or a two-tailed unpaired Student t test were used for statistical comparisons (* *p* < 0.05) using the Prism 6.0 software (GraphPad, San Diego, CA, USA).

## 3. Results

### 3.1. Composition of the Two Multistrain Preparations

The genetic identity of the two preparations was not investigated, since the composition was described in the package of each probiotic blend. However, it has to be noted that bacterial strains are coded in a different manner, which, as indicated in the package, suggests that the two preparations are made in different industrial plants and registered at different cell banks. Media and culture conditions were not described, and neither was the relative proportion of each bacterial strain in the final blend.

### 3.2. In Vitro Fermentation Activity 

Carbohydrate consumption causes a pH decrease, which is associated with the production of organic acid during fermentation. To test the fermentative pattern of Blend 1 and Blend 2, equal amounts of viable microorganisms from both preparations were grown in the presence of glucose or glucose and fructose at different concentrations, and the residual amount of each was evaluated after 18 h incubation. Blend 2 had a significantly greater effectiveness for fermenting glucose than its counterpart (median residual glucose 7.56 vs. 9.48 g/L, *p* = 0.000278) with similar amounts of growing microorganisms (median CFU: 1.57 × 10^9^ vs. 1.84 × 10^9^, *p* = ns). A similar effect was observed in the presence of fructose. Fructose was metabolized more efficiently by Blend 2 than by the other blend, and the residual amount of fructose after addition of 0.5% fructose was significantly lower with Blend 2 than with the counterpart (median residual fructose 2.09 vs. 4.11 g/L, *p* = 0.0198). Similar results were observed in the presence of 2% fructose (*p* = 0.0142). The increase in fructose utilization (consumption) was independent of changes in the growth of probiotic strains. In fact, with Blend 2 the microorganism count at the end of incubation reached a median value of 1.75 × 10^9^ and 1.55 × 10^9^ CFU/mL in the presence of fructose 0.5% and 2%, respectively, and 2.25 × 10^9^ and 2.38 × 10^9^ CFU/mL with Blend 1 (*p* = ns). The main metabolic product of carbohydrate fermentation was the production of organic acids, mainly lactic (Table 1 and Table 2). Thus, as shown in Table 1, despite similar bacterial growth and pH value (around 4.0), the two probiotic products showed a strong difference in the amount of lactic acid produced in response to the addition of 0.5% and 2.0% fructose (16.04 mg/mL vs. 13.85 mg/mL; *p* = 0.000046). Productions of both d- and l-stereo-isomeric forms of lactic acid were significantly higher in media inoculated with Blend 2 than with its counterpart (d-lactic acid, *p* = 0.0000028; l-lactic acid, *p* = 0.0048). Moreover, the difference in the production of d-lactic acid was significantly higher in comparison to the difference in the production of l-lactic acid. A similar effect was observed in presence of fructose.

### 3.3. d- and l-Lactic Acid Production Profiles by Probiotic Species 

Since production of the two stereo-isomeric forms of lactic acid varies from one species to another of lactic acid bacteria, we have investigated the formation capacity of individual species of Blend 2 by measuring the amount of d- and l-lactic acid produced after fermentation of glucose (basal medium). The results reported in Table 2 demonstrate that the production of the two isomers was highly different among *Lactobacillus* species. *L. plantarum* and *L. acidophilus* yielded substantial levels of both d- and l-lactic acid, while *L. bulgaricus* produced almost exclusively d-lactic acid and *L. paracasei* only l-lactic acid. *S. thermophilus* produced a good amount of l-lactic acid, while Bifidobacteria, *B. longum, B. breve*, and *B. infantis,* as expected, generated minimal amounts of the L isoform. 

### 3.4. Effects of the Probiotic Formulations in Mouse Models of Colitis

**Figure 1:**
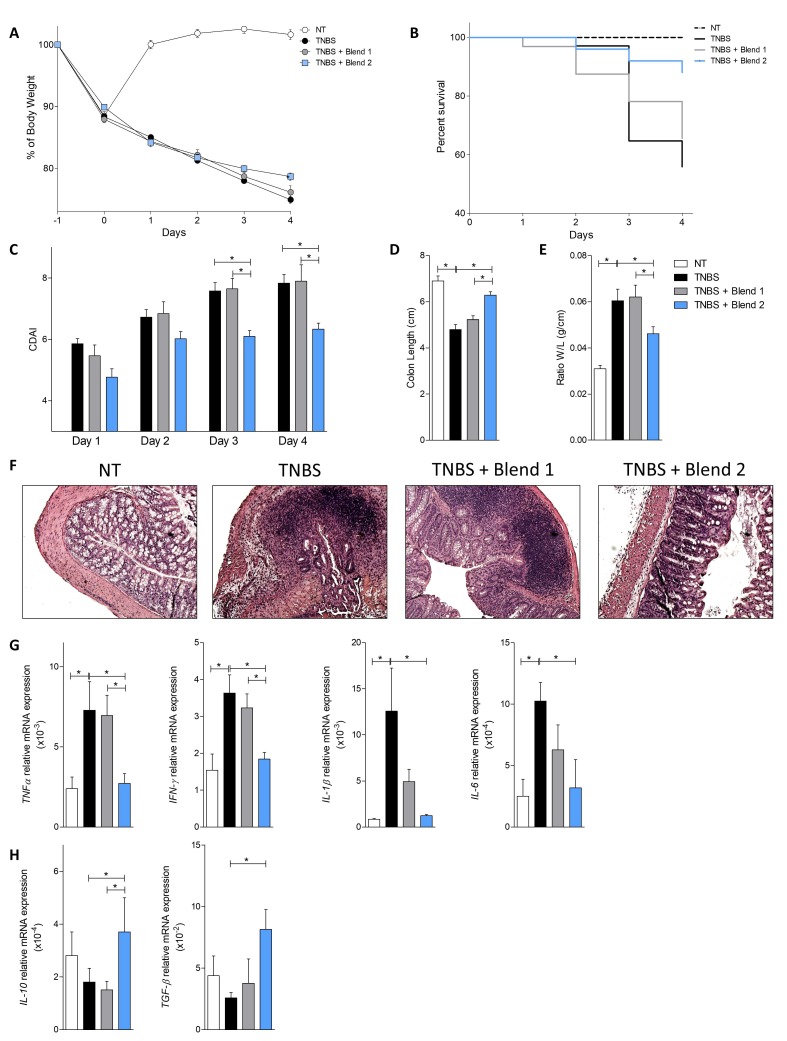
Effects of VSL#3 (Blend 1) and Vivomixx (Blend 2) on severity of colitis induced by 2,4,6-trinitrobenzenesulfonic acid (TNBS). Balb/c mice were injected intra-rectally as detailed in Materials and Methods with TNBS and then administered with vehicle or probiotics by gavage from day 0 to day 4. Blend 2 attenuated the development of the disease. Changes in body weight (**A**), survival (**B**), and the Colitis Disease Activity Index (CDAI) (**C**) of mice during the course of TNBS-induced colitis. Blend 2 reduced the intestinal inflammatory score: colon length (**D**) and ratio of colon weight/colon length (**E**). (**F**) H&E staining of colon sections from control mice, mice treated with TNBS, and mice treated with TNBS plus probiotics (original magnification 10×). Relative mRNA expression of Tnf-α, Ifn-γ, Il-1β, and Il-6 (**G**) and Il-10 and Tgf-β (**H**) genes in colon was assayed by real-time PCR. Data are normalized to Gapdh mRNA. Results are the mean ± SEM of 5–7 mice per group. * *p* < 0.05. Abbreviations are: interleukin (Il)-1β, -6, and -10; transforming growth factor (Tgf)-β; tumor necrosis factor (Tnf)-α; interferon (Ifn)-γ.

**Figure 2:**
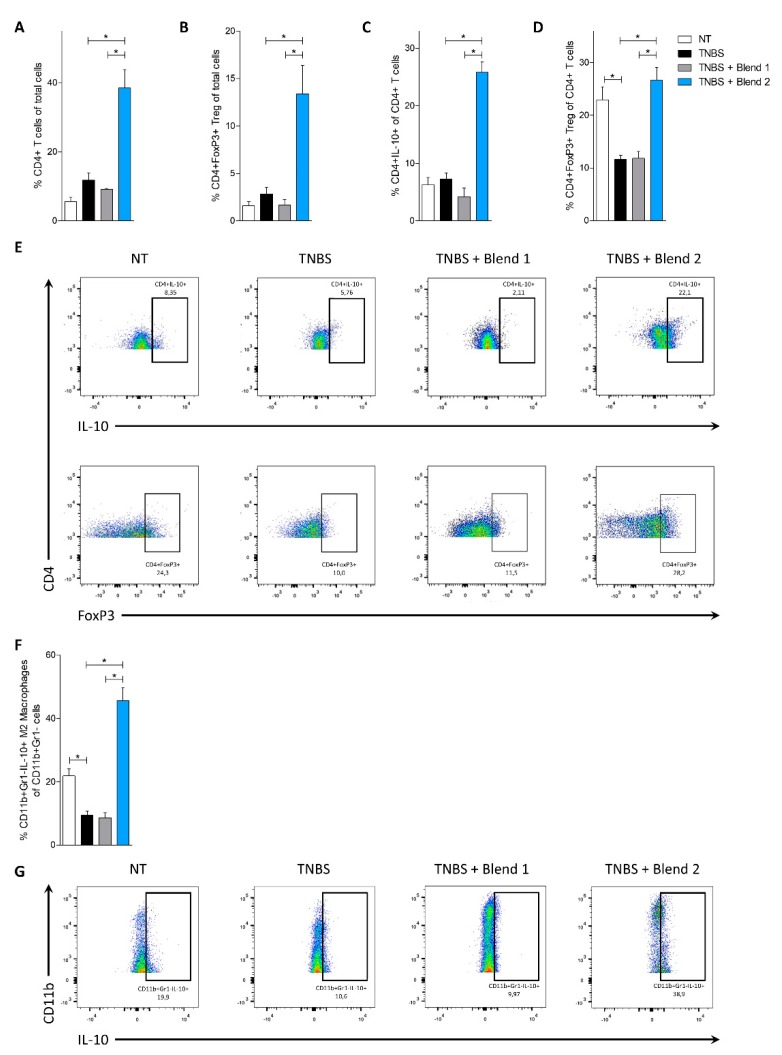
Vivomixx (Blend 2) but not VSL#3 (Blend 1) shapes the colonic *lamina propria* immune phenotype in the 2,4,6-trinitrobenzenesulfonic acid (TNBS) colitis. Balb/c mice were treated with TNBS as detailed in Materials and Methods and then administered with vehicle or one of the two formulations of probiotics by gavage from day 0 to day 4. Frequency of immune cells in the colonic *lamina propria*: CD4+ cells (**A**), CD4+FoxP3+ cells (**B**,**D**), and CD4+IL-10+ cells (**C**). (**E**) Flow cytometry analysis of IL-10 expression and FoxP3 expression in CD4+ cell recruited into *lamina propria*. (**F**) Frequency of CD11b+Gr1-IL-10+ M2 macrophages in the colonic lamina propria. (**G**) Flow cytometry analysis of Il-10 expression in CD11b+Gr1- macrophages recruited into the *lamina propria*. Results are the mean ± SEM of 5–7 mice per group. * *p* < 0.05. Abbreviations are: FoxP3, Forkhead box P3. Other abbreviations are listed in Figure 1.

We have therefore investigated the effect of the two probiotic formulations on two different models of murine colitis. The TNBS model of colitis is a widely used model of Th1-mediated disease, showing some similarities with Crohn’s disease (CD), while the model of DSS-induced colitis is considered a model for ulcerative colitis (UC). The severity of the TNBS-induced colitis, assessed by changes in body weight, survival, and the colitis disease activity index (CDAI) (Figure 1A–C), was attenuated by treatment with Blend 2, which also reduced mortality rate by approximately 30% and the CDAI by 75% at day 4, compared to the group treated with TNBS alone. In contrast, Blend 1 failed to achieve the same results. These findings were confirmed by the analysis of the macroscopic and microscopic characteristics of the colon (Figure 1D–F). In this setting, Blend 2 protected from the effects of TNBS on colon length, weight, and intestinal wall structure, while Blend 1 had no effect. Therefore, we have analyzed the expression of some pro- and anti-inflammatory cytokines in the colon of these mice. The data showed that the induction of colitis using TNBS induced an increase in the expression of pro-inflammatory genes such as Tnf-α, Inf-γ, Il-1β, and Il-6 (Figure 1G) and, on the other hand, a reduction in the expression of anti-inflammatory cytokines such as Il-10 and Tgf-β (Figure 1H). This inflammatory pattern was completely reversed by the administration of Blend 2, with a strong increase in the production of anti-inflammatory cytokines. In contrast, Blend 1 did not induce any effect on the expression of these genes compared to the group treated with TNBS alone. To better understand the effect of probiotic formulations on immune cells in the colon we extracted the *lamina propria* cells (LP) and performed a cytofluorimeter characterization (Figure 2). T lymphocyte analysis (Figure 2A–E) showed that the administration of Blend 2 induced a significant increase in the percentage of Treg cells (CD4^+^FoxP3^+^) and T lymphocytes producing the anti-inflammatory cytokine IL-10 (CD4^+^IL-10^+^) (Figure 2B–D). Further analysis of macrophages using flow cytometry (these cells are one of the most abundant leukocytes in the intestinal mucosa and are implicated in the pathogenesis of IBDs) showed that the administration of Blend 2 induced an increase in the polarization of macrophages to an M2 anti-inflammatory phenotype that produces IL-10 (Figure 2F,G). In contrast, the administration of Blend 1 had no effect on the polarization of both T lymphocytes and macrophages.

**Figure 3:**
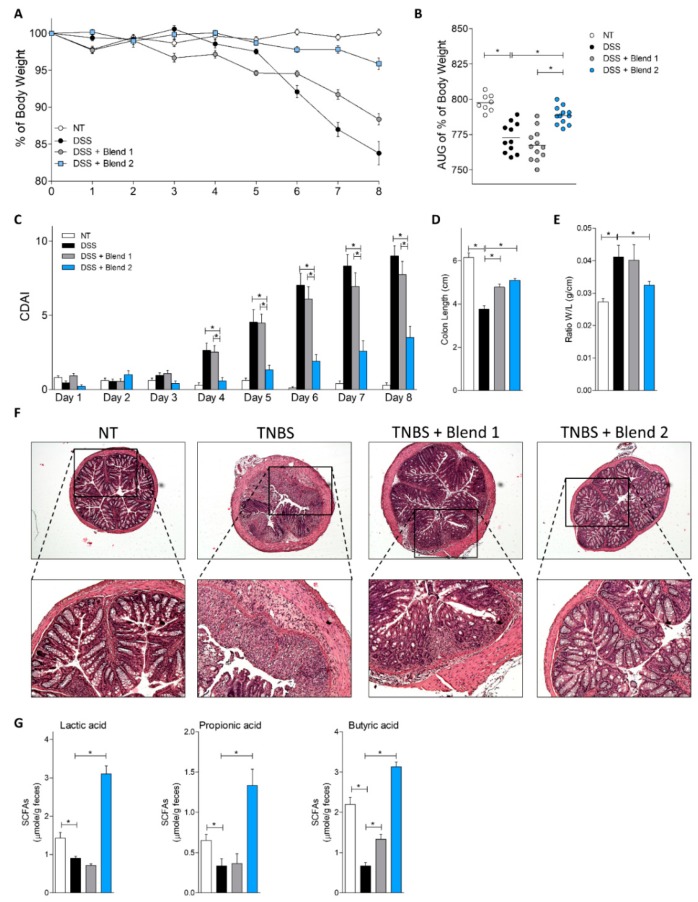
Effects of VSL#3 (Blend 1) and Vivomixx (Blend 2) on clinical course and severity on colitis induced by dextran sulfate sodium (DSS). Balb/c mice administered DSS in drinking water for 8 days alone or in combination with vehicle or one of the two probiotics by gavage from day 0 to day 8. Blend 2 attenuated the development of the disease. Changes in body weight (**A**), area under the curve (AUG) of % of body weight (**B**), and the Colitis Disease Activity Index (CDAI) (**C**) during the course of DSS-induced colitis. Blend 2 reduced the intestinal inflammatory score: colon length (**D**) and ratio of colon weight/colon length (**E**). (**F**) H&E staining of colon sections from control mice, mice treated with DSS, and mice treated with DSS plus probiotics (original magnification 10×). (**G**) Concentration of lactic acid (left panel), propionic acid (middle panel), and butyric acid (right panel) determined in the stool of mice collected on day 8. Results are the mean ± SEM of 8–12 mice per group. For panel B, each dot corresponds to a mouse. * *p* < 0.05. Abbreviations: SCFA, short-chain fatty acid.

**Figure 4:**
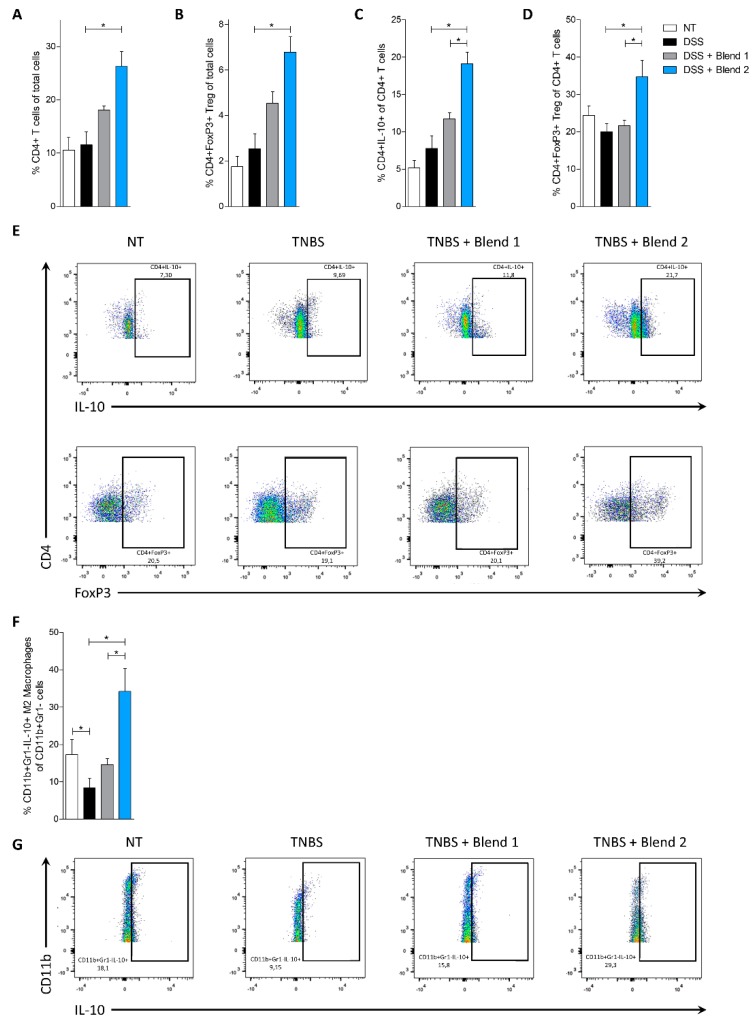
Effect of probiotics on the immune phenotype of colonic *lamina propria* cells in the model of colitis induced by dextran sulfate sodium (DSS). The experiment was carried out on Balb/c mice. Mice were treated with DSS in drinking water and then administered with vehicle or one of the two formulations of probiotics by gavage from day 0 to day 8. Frequency of immune cells in the colonic *lamina propria*: CD4+ cells (**A**), CD4+FoxP3+ cells (**B**,**D**), and CD4+IL-10+ cells (**C**). (**E**) Flow cytometry analysis of IL-10 expression and FoxP3 expression in CD4+ cell recruited into the *lamina propria*. (**F**) Frequency of CD11b+Gr1-IL-10+ M2 macrophages in the colonic *lamina propria*. (**G**) Flow cytometry analysis of Il-10 expression in CD11b+Gr1- macrophages recruited into the *lamina propria*. Results are the mean ± SEM of 6–10 mice per group. * *p* < 0.05. FoxP3, Forkhead box P3. Other abbreviations are listed in Figure 1.

To obtain further mechanistic insights on the effects of the two probiotic formulations, we have investigated whether similar effects were maintained in a second model of colitis induced by treating mice with DSS (Figure 3 and Figure 4). The analysis of the signs and symptoms of colitis assessed by body weight, CDAI, and macroscopic features of the colon (Figure 3A–E), showed that also in this model, administration of Blend 2 effectively reduced the severity of the disease, preventing weight loss induced by the DSS. Conversely, administration of Blend 1 failed to alleviate the development of colitis. These data were further confirmed by the histopathology analysis of the colon (Figure 3F). Furthermore, we found that Blend 2 administration effectively increased fecal content of lactic acid, propionic acid, and butyric acid. These compounds belong to the SCFAs produced at the intestine by the microbiota and are known for their ability to modulate formation and maturation of intestinal Treg. The data shown in Figure 3G demonstrate that treatment with DSS decreased the concentrations of all three mediators, and that this effect was completely reversed by Blend 2, whose administration effectively increased lactic acid and SCFA concentrations by two in comparison with naïve mice (*p* < 0.05). None of these effects was observed in mice treated with Blend 1. 

Taking into consideration that DSS exerts a profound inhibitory effect on the enzyme used in PCR analysis, we did not perform RT-PCR on the colon of these animals but instead carried out a flow cytometry analysis of the cells extracted from the LP (Figure 4). Consistent with results obtained in the TNBS model, we found that administration of Blend 2 (but not Blend 1) increased the percentage of CD4^+^Foxp3^+^ T cells (Figure 4A–E) and IL-10-producing macrophages (Figure 4F,G). None of these beneficial effects were observed in mice exposed to Blend 1.

### 3.5. The Effects of Probiotic Formulations on Intestinal Microbiota Composition 

**Figure 5:**
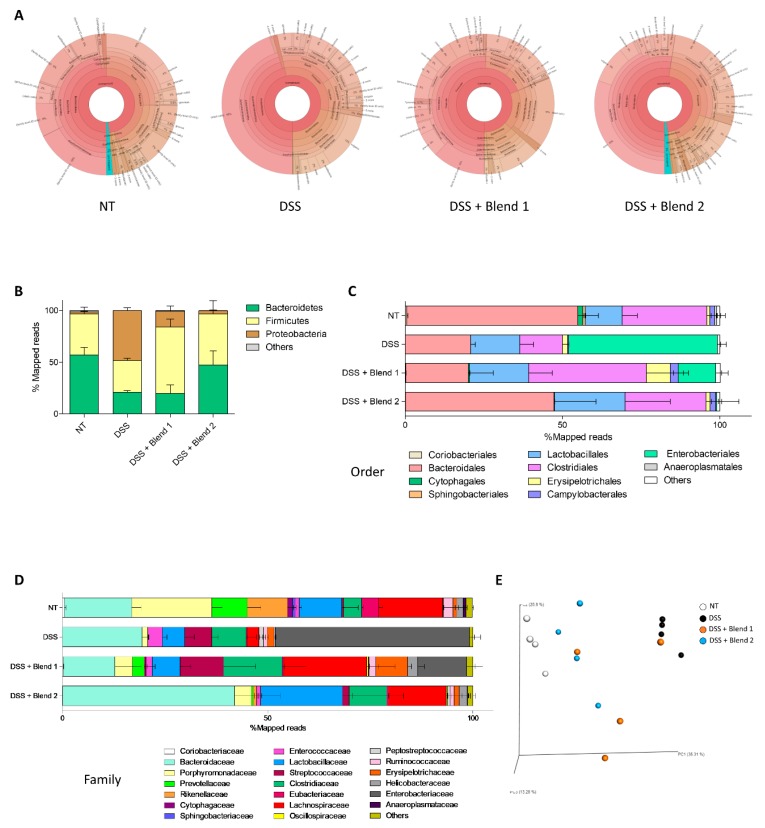
Effects of probiotic formulations on microbiome composition. The experiment was carried out on Balb/c mice. Mice were treated with DSS in drinking water and then administered with vehicle or one of the two formulations of probiotics by gavage from day 0 to day 8. (**A**) The visualization result of 16s analysis with taxonomy components of one representative sample of each group, using the Krona interface. (**B**) The gut microbiota composition profiles at phylum level, expressed as percentage of mapped reads. The gut microbiota composition profiles at order (**C**) and family (**D**) level, expressed as percentage of mapped reads. (**E**) The gut microbiota changes observed in mice differentiated by principal coordinate analysis (PCoA) using the Bray–Curtis dissimilarity matrices. Results are the mean ± SEM of 4 mice per group.

To obtain further insights into the impact of DSS and the two probiotic formulations on the intestinal microbiota, we then investigated the composition of the intestinal microbiota from four mice per group (Figure 5A) in fecal samples collected at the end of the study (eight days from start of colitis). Metagenomics analysis revealed a robust difference in microbiome composition expressed as relative abundance of phyla, calculated as percent of mapped reads. As displayed in Figure 5B, the DSS treatment radically changed the microbiome composition by reducing the relative percentage of *Bacteroidetes* (≈3 times, *p* < 0.005) and increasing the percentage of *Proteobacteria* (≈20 times, *p* < 0.0005). Both probiotic formulations modulated the microbiome composition compared to DSS treated mice, but the Blend 2 administration restored a phyla composition similar to that of untreated mice. There were no statistical differences in phyla between the “NT” group and the “DSS + Blend 2” group; while statistical differences remain in *Bacteroidetes* and *Proteobacteria* between the “NT” group and the “DSS + Blend 1” group (*p* < 0.05) (Figure 5B). 

We then assessed the relative abundance of bacterial orders, expressed as percentage of mapped reads (Figure 5C, Figures S1–S3). Exposure to DSS altered the composition of microbiota profoundly by increasing the percentage of *Enterobacteriales* and reducing the percentage of *Bacteroidales* and *Clostridiales* (Figure 5C). The data relative to the analysis of mice treated with Blend 1 and Blend 2, confirmed that both the probiotic preparations exerted an impact on gut microbiome composition, however, while Blend 2 reversed the effect of DSS by increasing the percentage of *Bacteroidales* and *Lactobacillales* and greatly reducing the percentage of *Enterobacteriales*, Blend 1 did not (Figure 5C). The gut microbiota composition was also analyzed at a more specific level, by calculating the relative abundance of bacterial family, expressed as a percentage of mapped reads (Figure 5D). The DSS administration notably increased the percentage of *Enterobacteriaceae* and *Streptococcaceae* family and reduced the percentage of other family, in particular that of *Lactobacillaceae* (Figure 5D). In mice treated with both the probiotic formulations, we have observed significant changes in family microbiota composition, however only the Blend 2 preparation completely reversed the DSS effect by reducing the percentage of *Enterobacteriaceae* and *Streptococcaceae* and restoring the percentage of *Lactobacillaceae* family (Figure 5D). These data were confirmed using quantitative β analysis of PCoA by family (Bray–Curtis analysis), which showed a minor dissimilarity between untreated mice and mice treated with Blend 2, while Blend 1 administration was significantly less effective in reversing the effect of DSS on family composition (Figure 5E). 

## 4. Discussion

The relevance of industrial production processes in modulating the functionality of probiotics in vivo is documented and we and others have recently provided evidence that different lots of the same product manufactured at different industrial sites, exert different in vitro and in vivo properties [7,17]. 

In the present study we have investigated the functionality of two multistrain preparations of probiotics in rodent models of colitis. As stated on their respective packages, the two preparations used in these studies are claimed to contain a blend of seven eight probiotic species. However, it should be noted that the two mixtures differ on several characteristics. First of all, that probiotic strains included in each preparation are labeled in a different manner suggesting a genetic diversity and/origin. Second, while both mixtures are reported to contain eight bacterial strains, the relative composition in terms of amount of each strain in the whole mixture is not known and might be different. Because individual probiotic strains perform different metabolic and immunological functions, variability in the amount of a specific strain in the final blend is likely to impact on mixture functionality. Third, the two preparations originate from different industrial sites and the composition of media used for industrial manufacturing are not known in detail, though one formulation (Blend 1) claims to be dairy-free, and this fact may introduce another robust source of variability. 

Beside the above-mentioned difference, the two probiotic mixtures differ in their functionality. In vitro investigations on the two blends demonstrated that the two mixtures differed significantly in their ability to produce d- and l-Lactic acid, and that the Blend 2 preparation generated a significantly higher amount of both d- and l-lactic acids than the counterpart. Because lactic acid is a part of the probiotic functionality and is a negative regulator of pro-inflammatory mediators such matrix metalloproteases, TNFα, CCL2 and CCL7, and NF-KB in monocytes [18,19], while inducing the expression of anti-inflammatory markers such as IL-23 [18,20] and EGR2 (a marker of M2 phenotype [20]), we speculated that the enhanced generation of d-and l-lactic acid by the Blend 2 formulation might contribute to its anti-inflammatory activity. Our in vitro analysis using individual probiotic species demonstrated that lactic acid is essentially produced, as expected, by the various Lactobacillus species, with a minor contribution by Bifidobacteria. Lactic acid is actively used by intestinal bacteria to generate propionate and butyrate, i.e., two of the best characterized bacterial metabolites in the gut [21]. Because the SCFAs are key bacterial metabolites and represent an essential component of probiotic functionality [22,23], we have examined whether these in vitro findings translate in vivo. Fittingly, results obtained in the DSS model of colitis demonstrated that while intestinal inflammation in DSS-treated mice resulted in a dramatic reduction of fecal content of lactate and two SCFAs, i.e., propionate and butyrate (Figure 3G), this pattern was reversed by treating mice with Blend 2 (i.e., the multistrain formulation that effectively released more lactate in vitro). Finally, since in vivo generation of lactate and butyrate in the intestine is also contributed to by Bacteroides species, the fact that Blend 2 administration reversed the Bacteroidetes reduction caused by DSS (Figure 5A) is further confirmation of the different functionality of the two blends [24,25].

Of relevance exposure to DSS resulted in a robust modification of intestinal microbiota with an increase in representation of *Proteobacteriaceae* and a concomitant reduction of *Bacteroides* without any significant changes in *Firmicutes* [26]. This finding confirms previous reports in various mice models of colitis, showing that progression of the disease is positively correlated to an increase of *Proteobacteria*, especially of the *Escherichia genus* [27]. The increase in facultative anaerobes, such as *Enterobacteriaceae*, in response to intestinal inflammation has been explained according to the “oxygen hypothesis” [28]. Thus, under normal conditions, colonic epithelial cells deplete oxygen levels in the lumen through beta-oxidation processes, generating an anaerobic environment. In the setting of local inappropriate and persistent inflammatory response the beta-oxidation capacity of the colonic epithelial cells decreases, leading to an increased availability of oxygen, which, in turn, promotes dysbiosis and is associated with increased *Proteobacteria*. Alternatively, it has been suggested that predominance of *Enterobacteriaceae* during inflammatory processes might be due the augmented availability of nitrates, produced by the host during inflammation, or the absence of butyrate-producing microbiota leading to a loss of PPAR-γ regulation of iNOS (inducible nitric oxide synthase) expression [29,30]. While the treatment with Blend 1 failed to impact on the structure of intestinal microbiota associated with DSS administration, the microbiological profile of the animals treated with Blend 2 was characterized by an appreciable similarity to what was observed in naïve animals assessed using principal coordinate analysis (PCoA) using the Bray–Curtis dissimilarity matrices. 

Because both blends claim efficacy in treating ulcerative colitis and ileal pouchitis, the functional effectiveness of the two multistrain preparations was examined in vivo in two rodent models of colitis. In both models, only treating mice with Blend 2 (Vivomixx) effectively reversed intestinal inflammation induced by TNBS and DSS. Results shown in Figure 1, Figure 2, Figure 3 and Figure 4, demonstrated that this blend significantly attenuated development of signs and symptoms of colitis, and that these beneficial effects associated with increased content of CD4^+^Foxp3^+^ cells and CD4^+^IL-10^+^ cells (Treg) in colon lamina propria. Thus, while the proportion of Treg was significantly reduced in mice treated with TNBS and DSS alone, this pattern was reversed by treating mice with Blend 2, while the other mixture failed to reach the same results. Treg cells are critical for maintaining immune homeostasis and establishing tolerance to non-pathogenic antigens, including commensal bacteria and food. These cells release suppressive cytokines, such as IL-10 and TGF-β, to regulate the balance of Th1/Th2 cells and maintain mucosal tolerance in the intestine [31,32]. Importantly, Treg formation is disabled in acute inflammation, and non-functional or absent Tregs or genetic mutations in Foxp3 induces hypersensitivity to bacterial antigens, altering the ability of the intestinal mucosal immunity to respond to inflammation [33]. In the present study, we found that the percentage of CD4^+^ Foxp3^+^ T cells (Figure 2D and Figure 4D) in the *lamina propria* was significantly reduced by treating mice with both TNBS and DSS, which also promoted a disturbance of Th1/Th2 cytokines balance and overexpression of pro-inflammatory mediators such as Il-1, Tnf-α, and Ifn-γ. This pattern was completely reversed by treating mice with Blend 2, which promoted a robust expansion of the number of CD4^+^ Foxp3 T cells and their signature cytokine, Il-10. In this study, the Blend 2 effectively increased the number of CD4^+^Foxp3^+^T cells and restored their suppressive function. Since SCFA play a critical role in controlling Treg generation and maturation [34,35,36], we speculated that Blend 2 alleviated inflammation and immune dysfunction by promoting a SCFA-dependent maturation of CD4^+^ Foxp3^+^T cells. 

In addition to SCFA, Blend 2 could modulate the formation of Foxp3+ Treg cells by restructuring the intestinal microbiota. For example, commensal *Bacteroides fragilis* polysaccharide promotes TLR2 dependent development of IL-10 producing Tregs and tolerance in experimental colitis [35]. Fittingly, as shown in Figure 5A, Blend 2 increased the amount of *Bacteroidetes* species in mice administered with DSS. 

One limitation of the present study is that we have investigated the functionality of the two probiotics in preclinical models of intestinal inflammation, and therefore the relevance of our results to humans need to be confirmed. Additionally, since the inflamed mice developed a specific microbiota, it is unclear whether effects observed with the two blends will extend to non colitic mice.

## 5. Conclusions

In summary, the results of the present study confirm that genomic label similarity does not guarantee the health promoting activities of the commercially available probiotics, and strongly advocate the need for better standardization of commercially available probiotic formulation not based exclusively genomics. 

## Figures and Tables

**Table 1:** Lactic acid production by probiotic blend grown in different growth medium. Blend 1 represents VSL#3 and Blend 2 represents Vivomixx.

Growth Medium	MRS			MRS + 0.5% Fructose			MRS + 2% Fructose		
Lactic acid (mg/mL) ^a^	d-	l-	Total	d-	l-	Total	d-	l-	Total
Blend 2	8.18 ± 0.33	7.86 ±0.77	16.04 ±0.97	8.70 ±0.72	8.16 ±0.76	16.86 ±1.39	8.67±0.90	8.22 ±0.87	16.90 ±1.64
Blend 1	6.96 ±0.56	6.89 ±0.58	13.85 ±0.98	6.41 ±0.53	6.27 ±0.70	12.67 ±1.20	7.03 ±0.78	6.83 ±0.74	13.86 ±1.36
*P* Blend 1 vs. Blend 2	0.0000028	0.0048	0.000046	0.0000001	0.0000086	0.0000004	0.00016	0.00058	0.00015

^a^ Each value represents the mean of at least three replicates from independent experiments.

**Table 2:** Lactic acid production by probiotic species.

Species	d-Lactic Acid	l-Lactic Acid	Total	CFU/mL ^a^
*L. plantarum*	8.29 ± 0.71	5.07 ± 0.39	13.36 ± 1.29	7.6 × 10^8^
*L. acidophilus*	3.91 ± 0.42	5.22 ± 0.52	9.13 ± 0.78	5.2 × 10^8^
*L. bulgaricus*	7.57 ± 0.69	0.24 ± 0.03	7.81 ± 0.72	6.3 × 10^9^
*L. paracasei*	0	7.19 ± 0.59	7.19 ± 0.59	8.6 × 10^8^
*S. thermophilus*	0	5.24 ± 0.46	5.24 ± 0.46	6.8 × 10^9^
*B. longum*	0	0.80 ± 0.05	0.80 ± 0.05	7.2 × 10^8^
*B. breve*	0	0.36 ± 0.06	0.36 ± 0.06	4.0 × 10^8^
*B. infantis*	0	0.34 ± 0.39	0.34 ± 0.39	5.9 × 10^8^

Values represent the mean concentration of lactic acid (mg/mL) produced in MRS broth (lactobacilli and bifidobacteria) or M17 broth (*S. thermophilus*) of two independent experiments. ^a^ Median CFU/mL after 21 h growth of individual strains inoculated at 3 × 10^5^ CFU/mL.

## Supplementary Materials

The following are available online at http://www.mdpi.com/2072-6643/11/2/325/s1, Figure S1: The visualization result of 16s analysis with taxonomy components of Proteobacteria phylum; Figure S2: The visualization result of 16s analysis with taxonomy components of Bacteroidetes phylum; Figure S3: The visualization result of 16s analysis with taxonomy components of Firmicutes phylum.

## References

[1] Food and Agriculture Organization of the United Nations (FAO)/World Health Organization (WHO) Guidelines for the Evaluation of Probiotics in Food. Proceedings of the Joint FAO/WHO Working Group on Drafting Guidelines for the Evaluation of Probiotics in Food.

[2] Hill C., Guarner F., Reid G., Gibson G.R., Merenstein D.J., Pot B., Morelli L., Canani R.B., Flint H.J., Salminen S. (2014). Expert consensus document. The International Scientific Association for Probiotics and Prebiotics consensus statement on the scope and appropriate use of the term probiotic. Nat. Rev. Gastroenterol. Hepatol..

[3] Marteau P., Shanahan F. (2003). Basic aspects and pharmacology of probiotics: An overview of pharmacokinetics, mechanisms of action and side-effects. Best. Pract. Res. Clin. Gastroenterol..

[4] Chapman C.M., Gibson G.R., Rowland I. (2011). Health benefits of probiotics: Are mixtures more effective than single strains?. Eur. J. Nutr..

[5] Sashihara T., Sueki N., Furuichi K., Ikegami S. (2007). Effect of growth conditions of Lactobacillus gasseri OLL2809 on the immunostimulatory activity for production of interleukin-12 (p70) by murine splenocytes. Int. J. Food Microbiol..

[6] van Baarlen P., Troost F.J., van Hemert S., van der Meer C., de Vos W.M., de Groot P.J., Hooiveld G.J., Brummer R.J., Kleerebezem M. (2009). Differential NF-kappaB pathways induction by Lactobacillus plantarum in the duodenum of healthy humans correlating with immune tolerance. Proc. Natl. Acad. Sci. USA.

[7] Biagioli M., Laghi L., Carino A., Cipriani S., Distrutti E., Marchianò S., Parolin C., Scarpelli P., Vitali B., Fiorucci S. (2017). Metabolic Variability of a Multispecies Probiotic Preparation Impacts on the Anti-inflammatory Activity. Front. Pharmacol..

[8] Cinque B., La Torre C., Lombardi F., Palumbo P., Van der Rest M., Cifone M.G. (2016). Production Conditions Affect the In Vitro Anti-Tumoral Effects of a High Concentration Multi-Strain Probiotic Preparation. PLoS ONE.

[9] Cinque B., La Torre C., Lombardi F., Palumbo P., Evtoski Z., Santini S., Falone S., Cimini A., Amicarelli F., Cifone M.G. (2017). VSL#3 probiotic differently influences IEC-6 intestinal epithelial cell status and function. J. Cell. Physiol..

[10] Trinchieri V., Laghi L., Vitali B., Parolin C., Giusti I., Capobianco D., Mastromarino P., De Simone C. (2017). Efficacy and Safety of a Multistrain Probiotic Formulation Depends from Manufacturing. Front. Immunol..

[11] Maassen C.B., Claassen E. (2008). Strain-dependent effects of probiotic lactobacilli on EAE autoimmunity. Vaccine.

[12] Mileti E., Matteoli G., Iliev I.D., Rescigno M. (2009). Comparison of the immunomodulatory properties of three probiotic strains of Lactobacilli using complex culture systems: Prediction for in vivo efficacy. PLoS ONE.

[13] Ríos-Covián D., Ruas-Madiedo P., Margolles A., Gueimonde M., De Los Reyes-Gavilán C.G., Salazar N. (2016). Intestinal Short Chain Fatty Acids and their Link with Diet and Human Health. Front. Microbiol..

[14] Mencarelli A., Cipriani S., Renga B., Bruno A., D’Amore C., Distrutti E., Fiorucci S. (2012). VSL#3 resets insulin signaling and protects against NASH and atherosclerosis in a model of genetic dyslipidemia and intestinal inflammation. PLoS ONE.

[15] Distrutti E., Cipriani S., Mencarelli A., Renga B., Fiorucci S. (2013). Probiotics VSL#3 protect against development of visceral pain in murine model of irritable bowel syndrome. PLoS ONE.

[16] Parise R.A., Beumer J.H., Kangani C.O., Holleran J.L., Eiseman J.L., Smith N.F., Covey J.M., Perrine S.P., Egorin M.J. (2008). Liquid chromatography-mass spectrometric assay for quantitation of the short-chain fatty acid, 2,2-dimethylbutyrate (NSC 741804), in rat plasma. J. Chromatogr. B Analyt. Technol. Biomed. Life Sci..

[17] Sanders M.E., Klaenhammer T.R., Ouwehand A.C., Pot B., Johansen E., Heimbach J.T., Marco M.L., Tennilä J., Ross R.P., Franz C. (2014). Effects of genetic, processing, or product formulation changes on efficacy and safety of probiotics. Ann. N. Y. Acad. Sci..

[18] Peter K., Rehli M., Singer K., Renner-Sattler K., Kreutz M. (2015). Lactic acid delays the inflammatory response of human monocytes. Biochem. Biophys. Res. Commun..

[19] Witkin S.S., Mendes-Soares H., Linhares I.M., Jayaram A., Ledger W.J., Forney L.J. (2013). Influence of vaginal bacteria and D- and L-lactic acid isomers on vaginal extracellular matrix metalloproteinase inducer: Implications for protection against upper genital tract infections. MBio.

[20] Witkin S.S., Alvi S., Bongiovanni A.M., Linhares I.M., Ledger W.J. (2011). Lactic acid stimulates interleukin-23 production by peripheral blood mononuclear cells exposed to bacterial lipopolysaccharide. FEMS Immunol. Med. Microbiol..

[21] Louis P., Hold G.L., Flint H.J. (2014). The gut microbiota, bacterial metabolites and colorectal cancer. Nat. Rev. Microbiol..

[22] Koh A., De Vadder F., Kovatcheva-Datchary P., Bäckhed F. (2016). From Dietary Fiber to Host Physiology: Short-Chain Fatty Acids as Key Bacterial Metabolites. Cell.

[23] Fiorucci S., Distrutti E. (2015). Bile Acid-Activated Receptors, Intestinal Microbiota, and the Treatment of Metabolic Disorders. Trends Mol. Med..

[24] Vital M., Howe A.C., Tiedje J.M. (2014). Revealing the bacterial butyrate synthesis pathways by analyzing (meta)genomic data. MBio.

[25] Million M., Tomas J., Wagner C., Lelouard H., Raoult D., Gorvel J.P. (2018). New insights in gut microbiota and mucosal immunity of the small intestine. Hum. Microb. J..

[26] Gronbach K., Flade I., Holst O., Lindner B., Ruscheweyh H.J., Wittmann A., Menz S., Schwiertz A., Adam P., Stecher B. (2014). Endotoxicity of lipopolysaccharide as a determinant of T-cell-mediated colitis induction in mice. Gastroenterology.

[27] Carvalho F.A., Koren O., Goodrich J.K., Johansson M.E., Nalbantoglu I., Aitken J.D., Su Y., Chassaing B., Walters W.A., González A. (2012). Transient inability to manage proteobacteria promotes chronic gut inflammation in TLR5-deficient mice. Cell Host Microbe.

[28] Rigottier-Gois L. (2013). Dysbiosis in inflammatory bowel diseases: The oxygen hypothesis. ISME J..

[29] Winter S.E., Winter M.G., Xavier M.N., Thiennimitr P., Poon V., Keestra A.M., Laughlin R.C., Gomez G., Wu J., Lawhon S.D. (2013). Host-derived nitrate boosts growth of *E. coli* in the inflamed gut. Science.

[30] Byndloss M.X., Olsan E.E., Rivera-Chávez F., Tiffany C.R., Cevallos S.A., Lokken K.L., Torres T.P., Byndloss A.J., Faber F., Gao Y. (2017). Microbiota-activated PPAR-γ signaling inhibits dysbiotic Enterobacteriaceae expansion. Science.

[31] Uhlig H.H., Coombes J., Mottet C., Izcue A., Thompson C., Fanger A., Tannapfel A. (2006). Characterization of Foxp3+CD4+CD25+ and IL-10-secreting CD4+CD25+ T cells during cure of colitis. J. Immunol..

[32] Liu B., Tonkonogy S.L., Sartor R.B. (2011). Antigen-presenting cell production of IL-10 inhibits T-helper 1 and 17 cell responses and suppresses colitis in mice. Gastroenterology.

[33] Mottet C., Uhlig H.H., Powrie F. (2003). Cutting edge: Cure of colitis by CD4+CD25+ regulatory T cells. J. Immunol..

[34] Bilate A.M., Bousbaine D., Mesin L., Agudelo M., Leube J., Kratzert A., Dougan S.K., Victora G.D., Ploegh H.L. (2016). Tissue-specific emergence of regulatory and intraepithelial T cells from a clonal T cell precursor. Sci. Immunol..

[35] Round J.L., Lee S.M., Li J., Tran G., Jabri B., Chatila T.A., Mazmanian S.K. (2011). The Toll-like receptor 2 pathway establishes colonization by a commensal of the human microbiota. Science.

[36] Smith P.M., Howitt M.R., Panikov N., Michaud M., Gallini C.A., Bohlooly Y.M., Glickman J.N., Garrett W.S. (2013). The microbial metabolites, short-chain fatty acids, regulate colonic Treg cell homeostasis. Science.

