# Supplementary figures and images for: Divergent Effectiveness of Multispecies Probiotic Preparations on Intestinal Microbiota Structure Depends on Metabolic Properties
Source: Nutrients. 2019 Feb 2;11(2):325. doi: 10.3390/nu11020325 (PMC6412585; doi:10.3390/nu11020325)

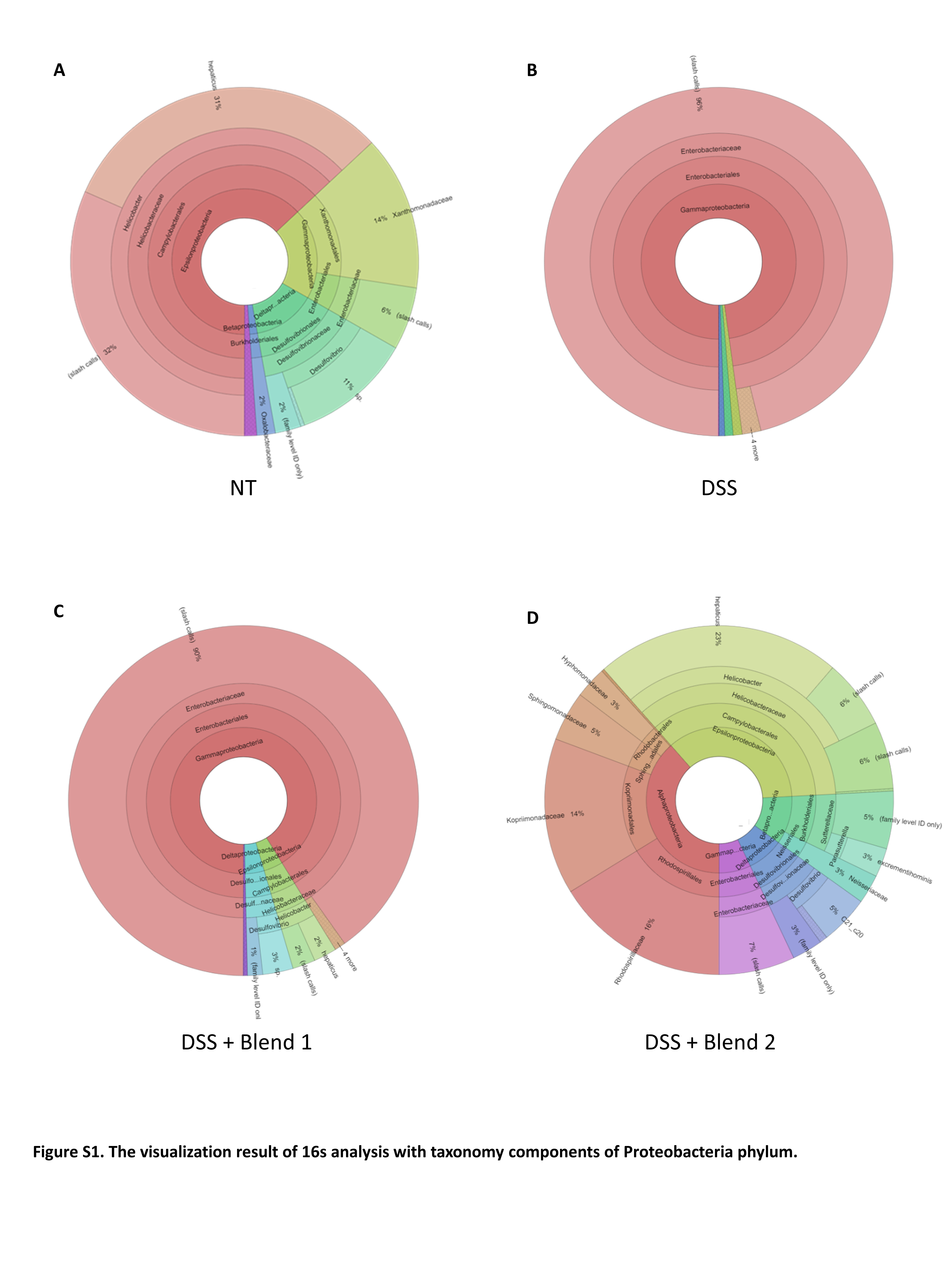

Supplement: Supplementary file 1 [file nutrients-11-00325-s001.zip › Figure S1.TIF]

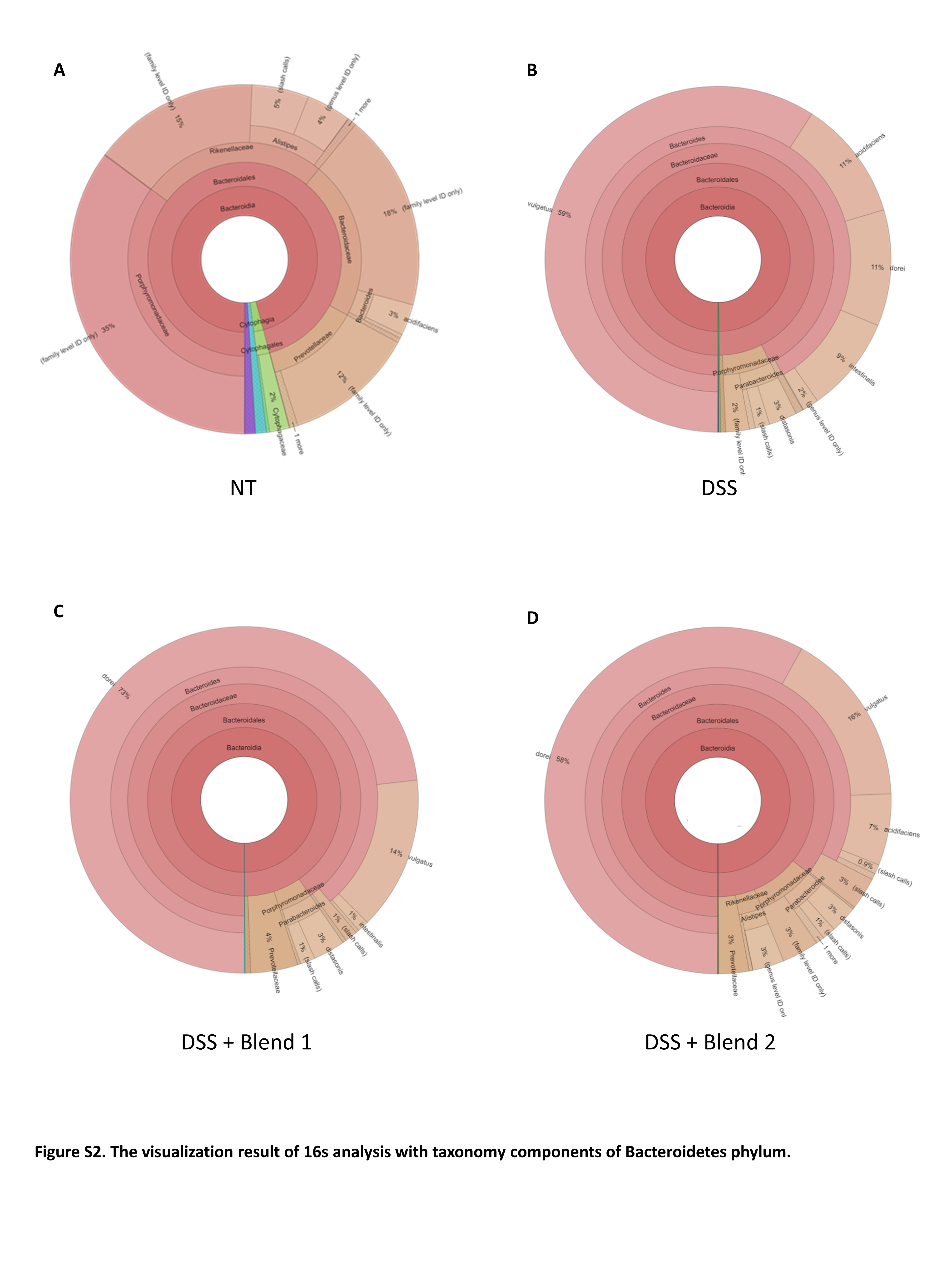

Supplement: Supplementary file 1 [file nutrients-11-00325-s001.zip › Figure S2.TIF]

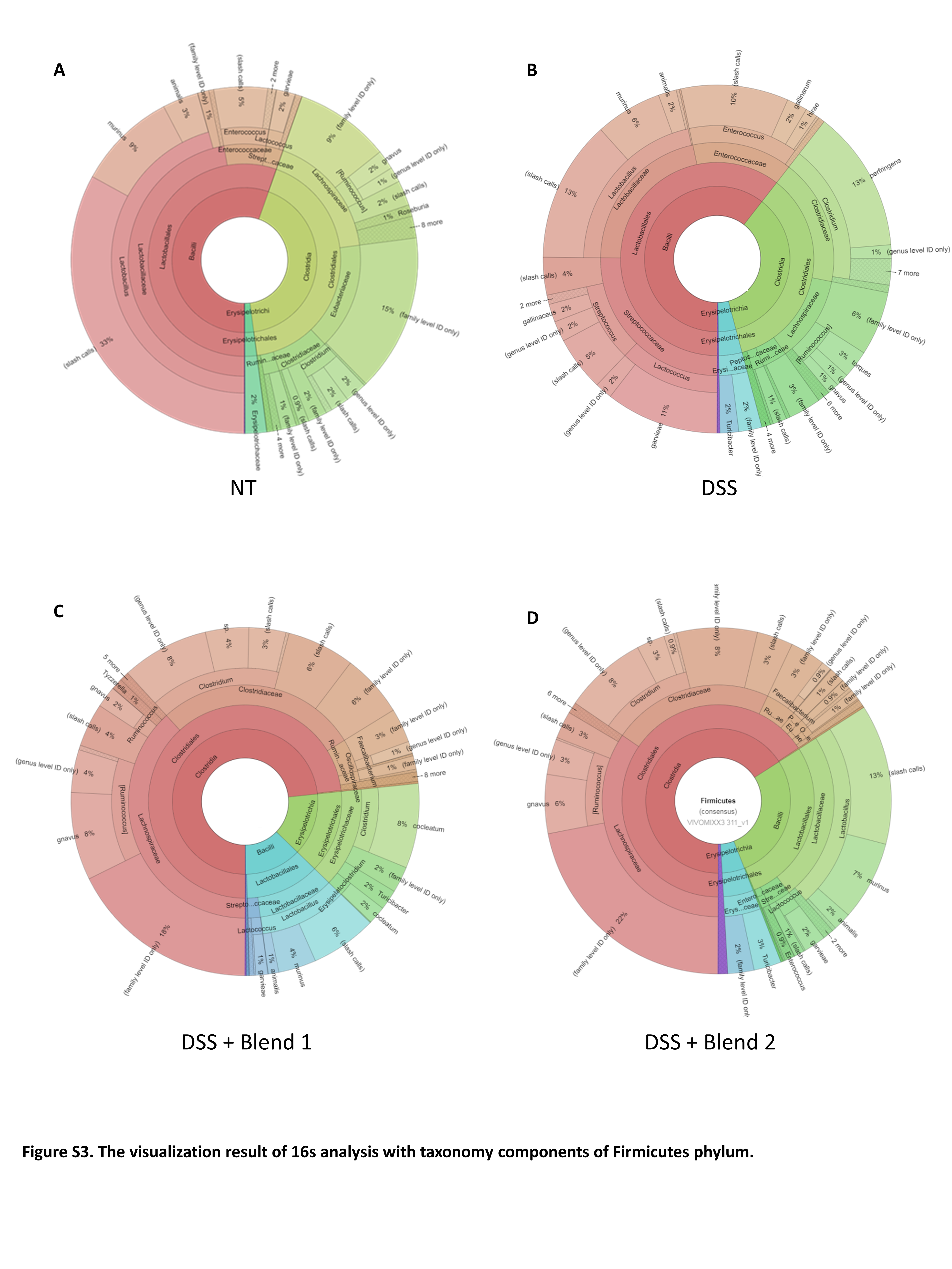

Supplement: Supplementary file 1 [file nutrients-11-00325-s001.zip › Figure S3.TIF]
